# The CannTeen Study: Cannabis use disorder, depression, anxiety, and psychotic-like symptoms in adolescent and adult cannabis users and age-matched controls

**DOI:** 10.1177/02698811221108956

**Published:** 2022-06-30

**Authors:** Will Lawn, Claire Mokrysz, Rachel Lees, Katie Trinci, Kat Petrilli, Martine Skumlien, Anna Borissova, Shelan Ofori, Catherine Bird, Grace Jones, Michael AP Bloomfield, Ravi K Das, Matthew B Wall, Tom P Freeman, H Valerie Curran

**Affiliations:** 1 Department of Psychology, Institute of Psychiatry, Psychology and Neuroscience, King’s College London, London, UK; 2 Department of Addictions, Institute of Psychiatry, Psychology and Neuroscience, King’s College London, London, UK; 3 Clinical Psychopharmacology Unit, University College London, London, UK; 4 Addiction and Mental Health Group (AIM), Department of Psychology, University of Bath, Bath, UK; 5 Department of Psychiatry, University of Cambridge, Cambridge, UK; 6 Department of Neuroimaging, Institute of Psychiatry, Psychology and Neuroscience, King’s College London, London, UK; 7 NIHR University College London Hospitals Biomedical Research Centre, University College Hospital, London, UK; 8 Centre for Affective Disorders, Institute of Psychiatry, Psychology and Neuroscience, King’s College London, London, UK; 9 Translational Psychiatry Research Group, Division of Psychiatry, Mental Health Neuroscience Department, University College London, London, UK; 10 Invicro London, London, UK

**Keywords:** Cannabis, marijuana, adolescence, depression, anxiety, addiction, cannabis use disorder, psychotic-like symptoms

## Abstract

**Background:**

Adolescence is characterised by psychological and neural development. Cannabis harms may be accentuated during adolescence. We hypothesised that adolescents would be more vulnerable to the associations between cannabis use and mental health and addiction problems than adults.

**Method:**

As part of the ‘CannTeen’ study, we conducted a cross-sectional analysis. There were 274 participants: split into groups of adolescent users (*n* = 76; 16–17 years old) and controls (*n* = 63), and adult users (*n* = 71; 26–29 years old) and controls (*n* = 64). Among users, cannabis use frequency ranged from 1 to 7 days/week, while controls had 0–10 lifetime exposures to cannabis. Adolescent and adult cannabis users were matched on cannabis use frequency (mean=4 days/week). We measured Diagnostic and Statistical Manual (DSM-5) Cannabis Use Disorder (CUD), Beck Depression Inventory, Beck Anxiety Inventory and Psychotomimetic States Inventory-adapted.

**Results:**

After adjustment for covariates, adolescent users were more likely to have severe CUD than adult users (odd ratio = 3.474, 95% confidence interval (CI) = 1.501–8.036). Users reported greater psychotic-like symptoms than controls (*b* = 6.004, 95% CI = 1.211–10.796) and adolescents reported greater psychotic-like symptoms than adults (*b* = 5.509, 95% CI = 1.070–9.947). User-group was not associated with depression or anxiety. No significant interactions between age-group and user-group were identified. Exploratory analyses suggested that cannabis users with severe CUD had greater depression and anxiety levels than cannabis users without severe CUD.

**Conclusion:**

Adolescent cannabis users are more likely than adult cannabis users to have severe CUD. Adolescent cannabis users have greater psychotic-like symptoms than adult cannabis users and adolescent controls, through an additive effect. There was no evidence of an amplified vulnerability to cannabis-related increases in subclinical depression, anxiety or psychotic-like symptoms in adolescence. However, poorer mental health was associated with the presence of severe CUD.

## Introduction

Adolescence is considered to be a dynamic period that begins with puberty and ends when one achieves an independent role in society (Dumontheil, 2016). Brain structure, brain function, neurotransmitter systems (including the endocannabinoid system), cognitive function and emotional processing continue to mature during adolescence (Blakemore and Choudhury, 2006; Galve-Roperh et al., 2009; Giedd et al., 1999; Shaffer, 1996; Smetana and Villalobos, 2009). Mental health problems typically emerge during adolescence, with 50% beginning before the age of 18 years and 75% by the age of 24 years (Jones, 2013; Kessler et al., 2005; Kieling et al., 2011; Patel et al., 2007; Paus et al., 2008). No agreed-upon age range constitutes ‘adolescence’, but definitions are usually 10–19 years or 10–24 years (Sawyer et al., 2018).

Cannabis is the most commonly used internationally-controlled recreational psychoactive substance in the world with 3.9% of the world’s population reporting use in the past year (UNODC, 2020). Cannabis is particularly popular in adolescents, with 28.0% of 15- to 16-year olds in the United States (NIDA, 2020) and 19.3% of 15-year olds in England reporting use in the past year (NHS-Digital, 2018, 2019). In 2019, cannabis was the primary problem drug for 77% of young people (aged under 18 years) in England who received treatment for alcohol or illicit drug problems (NDTMS, 2019).

Concerns have been raised about the disruptive effect adolescent drug use can have during this crucial developmental stage in one’s life (Blest-Hopley et al., 2020; Levine et al., 2017; Lubman et al., 2007; Volkow et al., 2018). Theoretically, adolescents endure substantially greater cannabis-induced harm than their adult counterparts because their brains are more malleable and vulnerable to the drug’s impacts (Dow-Edwards and Silva, 2017).

Cannabis use carries a risk of addiction. Results from large, representative epidemiological studies in the United States from the 1990s and 2000s showed that roughly 9% of people who try cannabis transition to dependence at some point in their life (Anthony et al., 1994; Lopez-Quintero et al., 2011). However, Leung et al. (2020) conducted a meta-analysis of studies from 2009 onwards and found that among people who had tried cannabis, 22% developed a cannabis use disorder (CUD) (Leung et al., 2020), while approximately 30% of last-year users in the United States have a CUD (Hasin et al., 2015). Younger current age and age of first cannabis use (age-of-onset) reliably augment the risk of developing CUD (Chen et al., 2005, 2009; Ehlers et al., 2010; Le Strat et al., 2015; Leung et al., 2020; Lopez-Quintero et al., 2011; Von Sydow et al., 2002; Wagner and Anthony, 2002; Wittchen et al., 2011). These studies tend to converge on an odds ratio (OR) of approximately 3 for the risk of CUD in adolescents compared to adults. However, these studies typically failed to match or account for cannabis use frequency differences between adolescents and adults, which is strongly related to addiction severity (Freeman and Winstock, 2015).

Results from multiple large-scale studies indicate that there is a small association between cannabis use and depression (Degenhardt et al., 2001, 2003; Horwood et al., 2012). A meta-analysis of longitudinal studies found cannabis users had a higher likelihood of developing depression compared to controls (OR = 1.17) (Lev-Ran et al., 2014) and another meta-analysis supported the link between cannabis use in adolescence and depression in early adulthood (OR = 1.37) (Gobbi et al., 2019). Some longitudinal studies have reported a greater vulnerability to depression in those with a younger age-of-onset (Horwood et al., 2012; Schoeler et al., 2018) and one systematic review concluded that there was some evidence that a younger age-of-onset was linked to depression (Hosseini and Oremus, 2019). However, a nonlinear association between age and contemporaneous depressive symptoms has been reported (Leadbeater et al., 2019). Moreover, a meta-analysis did not find a significant age-of-onset effect (Lev-Ran et al., 2014). Hence, an age-specific vulnerability to cannabis-related depression is unclear. Moreover, the population cohort studies that contribute to these meta-analyses often include small numbers of frequent cannabis users and define frequent cannabis liberally, for example, once per month (Pedersen, 2008; Schoeler et al., 2018). Purposive sampling of frequent cannabis users is one way to address this.

In a cross-sectional survey, Crippa et al. (2009) found higher levels of anxiety symptoms in people who frequently use cannabis compared to controls (Crippa et al., 2009), while Troisi et al. (1998) found greater levels of cannabis use were associated with greater severity of anxiety symptoms (Troisi et al., 1998). In a meta-analysis of longitudinal studies, Xue et al. (2020) reported that, overall, cannabis use increased odds of developing any future anxiety condition (OR = 1.25) (Xue et al., 2020). However, Xue et al. (2020) concluded that results varied considerably, which may be due to differences in cannabis exposure and analytical differences. Crucially, the evidence for a relationship between early age-of-onset and later symptoms of anxiety is inconclusive (Fergusson and Horwood, 1997; Gage et al., 2015; Guttmannova et al., 2017). Furthermore, the association between contemporaneous cannabis use and anxiety during adolescence and adulthood has not been researched.

Longitudinal studies have consistently reported an association between cannabis use and an increased risk of psychosis and schizophrenia (Andréasson et al., 1987; Hjorthøj et al., 2021; Marconi et al., 2016; Moore et al., 2007; Van Os et al., 2002; Zammit et al., 2002). Cannabis use during adolescence, compared to adulthood, has been associated with a greater risk of psychotic outcomes (Di Forti et al., 2014; Dragt et al., 2012; Galvez-Buccollini et al., 2012; Large et al., 2011; Manrique-Garcia et al., 2012; Schimmelmann et al., 2011). However, a large cross-sectional study found that in adolescents and young adults, current cannabis use was only associated with psychotic symptoms *after* the age of 22 years and that there was no relationship with age-of-onset (Leadbeater et al., 2019). Positive associations have also consistently been reported between cannabis use and psychotic-like experiences or subclinical symptoms of psychosis (Arseneault et al., 2002; Fergusson et al., 2003; Henquet et al., 2005; Hides et al., 2009; Kuepper et al., 2011; Miettunen et al., 2008; Stefanis et al., 2004). Moreover, earlier age-of-onset has been linked with greater psychotic-like symptoms in later life in two studies (Schubart et al., 2011; Stefanis et al., 2004) and cannabis use during adolescent has been associated with psychotic-like symptoms 1 or 2 years afterwards (Bourque et al., 2018). Additionally, the relationship between current cannabis use and concurrent psychotic-like symptoms in adolescents and adults is unknown.

As reviewed above, there are theoretical and empirical grounds for suggesting that earlier, adolescent use of cannabis may be particularly deleterious to mental health. However, there remain large variations in study design, disparate measures of cannabis use and mental health and a plethora of discrepant findings regarding age-specific vulnerability. Few studies have compared the contemporary mental health of adolescents with adults, while adolescents are still under 18 years old. Whether adolescent cannabis use, compared to adult use, genuinely heightens the risk of poor mental health remains an unanswered question. In this study, we therefore compared how current cannabis use may be associated with the presence of severe CUD, and the severity of subclinical depression, anxiety, and psychotic-like symptoms in adolescents and adults. Our cannabis-using groups were matched on current cannabis use frequency and our age-groups were matched on gender and age. Adult users had not frequently used cannabis before the age of 18 years.

As registered on the Open Science Framework (Lawn et al., 2021), and on the basis of evidence reviewed here, our hypotheses were as follows:

1. Adolescent users will be more likely than adult users to have severe CUD.
2. Cannabis users will have higher levels of (a) depression, (b) anxiety and (c) psychotic-like symptoms than controls.
3. There will be an interaction between user-group and age-group on (a) depression, (b) anxiety and (c) psychotic-like symptoms such that the difference between users and controls (where users > controls) will be greater in adolescents than adults.

For each hypothesis, we also predicted that the association would persist after adjusting for covariates. In addition, we conducted exploratory, unregistered analyses investigating the relationship between severe CUD and mental health symptoms.

## Methods

### Study design

This analysis uses cross-sectional, baseline data from the ‘CannTeen’ longitudinal project. The design has two between-subjects factors: age-group (adolescents and adults) and user-group (users and controls). The full study protocol (Lawn et al., 2020) describes overall aims, participants, power analysis, data collection procedures, tasks and timelines. Ethical approval was obtained from the University College London ethics committee, project ID 5929/003. The study was conducted in line with the Declaration of Helsinki, and all participants provided written, informed consent.

### Participants

The full sample comprises 274 participants: 76 adolescent users, 71 adult users, 63 adolescent controls and 64 adult controls.

Participants were recruited using online advertisements on Facebook, Instagram, Gumtree, and Reddit; school assemblies in London and the surrounding area; in-person flyering; and word-of-mouth. We recruited participants in a targeted process, by advertising to specific age-groups. Potential participants were screened and selected based on their cannabis use and other criteria. Participants were compensated financially for their time (£240 for completing all sessions with payments split across five separate sessions over a 12-month period).

For full eligibility criteria, see the Supplemental Materials. In brief, adolescents were aged 16–17 years and adults aged 26–29 years; users reported using cannabis recreationally between 1 and 7 days per week; adult users were excluded if they had used cannabis on a weekly or more frequent basis before the age of 18 years; and controls reported using either cannabis or tobacco at least once in their life, but with no more than 10 lifetime uses of cannabis. We recruited controls with limited cannabis or tobacco exposure, rather than people with no exposure, with the aim of more closely matching the controls and users on the opportunity to use drugs and associated unmeasurable confounders.

Exclusion criteria for all participants were as follows: current daily use of psychotropic medication, current treatment for a mental health disorder including CUD, a personal history of psychotic disorder, or use of any illicit drug except cannabis more than twice per month.

### Measures

#### Exposure variables

##### Age-group

Participants were either adults (aged 26–29 years; coded as 0) or adolescents (aged 16–17 years; coded as 1).

##### Cannabis use frequency

Using a timeline follow-back (TLFB) (Robinson et al., 2014) method, we measured cannabis use frequency in days/week over the previous 12 weeks.

#### Outcome variables

##### *Beck Depression Inventory-II* (Beck and Brown, 1996)

A 21-item self-report questionnaire. Each item is answered with ‘not at all’, ‘mildly’, ‘moderately’ or ‘severely’ and scored from 0 to 3, with total scores ranging from 0 to 63. Higher scores indicate greater levels of depression.

##### *Beck Anxiety Inventory* (Beck et al., 1988)

A 21-item self-report questionnaire. Each item is scored from 0 to 3, with total scores ranging from 0 to 63. Higher scores indicate greater levels of anxiety.

##### *Psychotomimetic States Inventory-Adapted* (Mason et al., 2008)

The Psychotomimetic States Inventory-Adapted (PSI-a) is a temporally adapted version of the original PSI, a 48-item self-report questionnaire assessing psychotic-like symptoms. Participants were asked questions about how they felt over ‘the last 2 weeks’, rather than ‘right now’. Each item is answered with ‘not at all’, ‘slightly’, ‘moderately’ or ‘strongly’ and scored from 0 to 3. Total scores range from 0 to 144. Higher scores indicate greater psychotic-like symptomatology.

*Diagnostic and Statistical Manual of Mental Disorders Fifth Edition (DSM-5) CUD* (American Psychiatric Association, 2013) *using the Mini International Neuropsychiatric Interview* (Sheehan et al., 1998)

Severity of CUD was assessed using the Mini International Neuropsychiatric Interview, in which 11 Diagnostic and Statistical Manual of Mental Disorders Fifth Edition (DSM-5) symptoms are assessed over the last 12 months. The presence of 0–1 symptoms is considered ‘none’, 2–3 symptoms is considered ‘mild’, 4–5 symptoms is considered ‘moderate’, and 6 or more symptoms is considered ‘severe’. We categorised users into having ‘severe CUD’ (coded as 1) or ‘not severe CUD’ (coded as 0).

##### Pre-defined covariates

We adjusted for gender, risk-taking, socioeconomic status (SES), problem alcohol use, tobacco use (non-cannabis related), and other illicit drug use. Risk-taking was measured using the total score from the RT-18 questionnaire (De Haan et al., 2011). SES was dichotomously measured using maternal education level, with categories of below undergraduate degree or undergraduate degree and above. Problematic alcohol use was measured using the total score from the alcohol use disorder identification test (Babor et al., 2001). Daily (non-cannabis) tobacco use was dichotomously measured using the TLFB, with categories of daily (⩾6.5 days per week) or non-daily (<6.5 days per week) tobacco smoking. Other illicit drug use was dichotomously measured using the TLFB, with categories of monthly (⩾1 day per month) or less than monthly (<1 day per month). See Supplemental Materials for description of these variables.

### Procedure

As per the full protocol (Lawn et al., 2020), participants were first screened and then potentially eligible participants were invited to a baseline session. At the start of the baseline session, inclusion and exclusion criteria and study-required abstinence (zero breathalyser reading; negative saliva drugs screen; self-reported alcohol and cannabis abstinence for 12 hours; self-reported other illicit drug use abstinence for 48 hours) were checked. Subsequently, participants completed the session including the measures described above.

### Power

We powered the study to detect a cross-sectional group difference in CUD between adolescent and adult cannabis users, as this is a robust finding with a quantified effect size (Chen et al., 2005; Ehlers et al., 2010; Le Strat et al., 2015) of OR = 3, equivalent to Cohen’s *d* = 0.6 or Cohen’s *f* = 0.3. With *ɑ* = 0.05 and a desired power of 0.95, 148 users were required, split evenly between adolescent and adult users. Crucially, in terms of detecting age-group by user-group interactions, with our total sample size (*n* = 274) and an assumed power of 0.8 we are powered to detect at least small–medium interactions of size Cohen’s *f* ⩾ 0.17 (Cohen’s *f* effect size around 0.1 are considered small, *f* effect size around 0.25 is considered medium and *f* effect size around 0.4 is considered large).

### Statistical analyses

Analyses were pre-registered on the Open Science Framework (Lawn et al., 2021). Statistical tests were conducted on IBM SPSS Statistics Version 27. For pre-processing of data, assumptions of analyses and details of missing data, see the Supplemental Materials. We ran linear and logistic regression models in a block-wise manner, see Supplemental Table S4. Models first included user-group, then age-group and user-group, then their interaction, and then we added pre-defined covariates to the best previous model (which was invariably the model with user-group and age-group as main effects, never the model with the interaction). We used an alpha value of 0.05. We ran post-hoc Bayesian *t*-tests to assess the null findings for users versus controls, and for adolescent users versus adult users, with no adjustment for covariates. We assumed equal variances and used a Jeffreys default prior. Bayes factors (BF_01_) ⩾ 3 support the null hypothesis of no difference.

Exploratory, unregistered analyses were conducted to investigate the relationships between the presence of severe CUD and Beck Depression Inventory (BDI), Beck Anxiety Inventory (BAI), and PSI-a (see Supplemental Materials for full details).

## Results

### Participant characteristics (Table 1)

All groups had a similar number of males and females. Adolescent users (3.7 days/week) and adult users (4.1 days/week) were matched on cannabis use frequency (*t*_145_ = 1.198, *p* = 0.233, *d* = 0.198). The time since last cannabis use did not differ between adolescent users (2.4 days) and adult users (2.5 days) (*t*_145_ = 0.118, *p* = 0.906, *d* = 0.019). Furthermore, a similar number of adolescent users (*n* = 69, 90.8%) and adult users (*n* = 59, 83.1%) used strong herbal cannabis as their most common type of cannabis, and these distributions did not differ significantly ( $\chi_{3}^{2}=3.866$ , *p* = 0.276). However, adolescents reported using more cannabis (1.1 g) on a day of use than adults (0.6 g) (*t*_142_ = 3.623, *p* < 0.001, *d* = 0.605). See Table 2 for data on cannabis use variables. Adolescent users (17.1 years) and adolescent controls (17.1 years) were matched on age (*t*_137_ = 0.224, *p* = 0.823, *d* = 0.038), as were adult users (27.6 years) and adult controls (27.4 years) (*t*_145_ = 1.232, *p* = 0.220, *d* = 0.212). See Table 1 and Supplemental Materials for differences in demographic variables.

**Table 1.** Summary of participant demographics.

	Adolescent control (***n*** = 63)	Adolescent user (***n*** = 76)	Adult control (***n*** = 64)	Adult user (***n*** = 71)	Group differences
**Gender**					
Male	31 (49.2%)	38 (50.0%)	31 (48.4%)	38 (53.5%)	
Female	32 (50.8%)	38 (50.0%)	33 (51.6%)	33 (46.5%)	
Age (years)					
	17.1 (0.5) [17.1, 16.1–18.0	17.1 (0.5) [17.1, 16.2–18.0]	27.4 (1.0) [27.3, 26.0–30.0]	27.6 (1.2) [27.3, 26.0–30.0]	Adults > adolescents***
Ethnicity					
White	40 (63.5%)	51 (68.0%)	41 (64.1%)	45 (63.4%)	
Mixed	7 (11.1%)	15 (20.0%)	3 (4.7%)	8 (11.3%)	
Asian	10 (15.9%	2 (2.7%)	15 (23.4%)	11 (15.5%)	
Black	2 (3.2%)	4 (5.3%)	2 (3.1%)	6 (8.5%)	
Other	2 (3.2%)	3 (4.0%)	2 (3.1%)	1 (1.4%)	
Prefer not to say	2 (3.2%)	0 (0.0%)	1 (1.6%)	0 (0.0%)	
SES					
Mother’s education below undergraduate degree	26 (41.9%)	31 (41.3%)	36 (57.1%)	37 (54.4%)	
Mother’s education undergraduate degree or above	36 (58.1%)	44 (58.7%)	27 (42.9%)	31 (45.6%)	Adolescents > adults *
RT-18	9.1 (4.1) [10.0, 0.0–17.0]	11.4 (3.1) [11.0, 3.0–18.0]	7.6 (4.1) [7.0, 0.0–16.0]	8.8 (3.9) [8.0, 3.0–17.0]	Users > controls*** and Adolescents > adults***
Alcohol, use frequency (days/week)	0.7 (0.8) [0.4, 0.0–3.7]	0.6 (0.6) [0.4, 0.0-3.3]	1.4 (1.0) [1.4, 0.0–5.3]	1.5 (1.4) [0.9, 0.0–6.8]	Adults > adolescents***
AUDIT	4.3 (3.5) [4.0, 0.0–13.0]	6.5 (4.6) [6.0, 0.0–18.0]	5.5 (4.2) [5.0, 0.0–22.0]	6.0 (4.3) [5.0, 0.0–18.0]	Users > controls **
Tobacco, daily use					
No	61 (96.8%)	66 (86.8%)	62 (96.9%)	62 (87.3%)	
Yes	2 (3.2%)	10 (13.2%)	2 (3.1%)	9 (12.7%)	Users > controls **
Other illicit drug use, monthly use					
No	61 (96.8%)	31 (40.8%)	63 (98.4%)	53 (74.6%)	
Yes	2 (3.2%)	45 (59.2%)	1 (1.6%)	18 (25.4%)	Users > controls*** and Adolescent users > adult users***

Sociodemographic and non-cannabis drug use variables for adolescent controls, adolescent users, adult controls and adult users. Group differences are highlighted in the final column. SES (maternal education) has data missing for one adolescent user, one adolescent control, three adult users and one adult control. Ethnicity has one adolescent user missing. Alcohol frequency and AUDIT scores include participants who have not consumed alcohol within the last 12 weeks and they are assigned zero. For continuous data mean (SD) [median, minimum–maximum] is shown, for categorical data *n* (%) is shown.

AUDIT: alcohol use disorders identification test; RT-18: Risk-Taking 18; SD: standard deviation; SES: socioeconomic status.

* *p* < 0.05, **p < 0.01, ***p < 0.001.

**Table 2.** Cannabis use variables for adolescent controls, adolescent users, adult controls and adult users.

	Adolescent control (*n* = 63)	Adolescent user (*n* = 76)	Adult control (*n* = 64)	Adult user (*n* = 71)	Group differences
Ever used cannabis (controls)					
No	8 (12.7%)	NA	2 (3.1%)	NA	
Yes	55 (87.3%)	NA	62 (96.9%)	NA	Adult controls > adolescent controls*
Cannabis, number of lifetime uses (controls)	3.4 (2.8) [2.0, 0.0–10.0]	NA	4.5 (3.1) [4.0, 0.0–10.0]	NA	Adult users > adolescent users*
Cannabis, time since last use (days) (users)	NA	2.4 (2.6) [1.6, 0.5–14.0]	NA	2.5 (4.6) [1.0, 0.5–35.0)	
Cannabis, age of first ever use (years) (users)	NA	14.6 (1.1) [14.7, 11.0–16.6]	NA	18.0 (2.9) [17.9, 13.0–25.0]	Adult users > adolescent users*
Cannabis, use frequency (days/week) (users)	NA	3.7 (2.0) [3.4, 0.8–6.9]	NA	4.1 (1.9) [3.8, 0.8–6.9]	
CUDIT-R	NA	15.4 (5.6) [5.0–27.0]	NA	11.9 (4.8) [3.0–26.0]	Adolescent users > adult users***
Number of users who most commonly use strong herbal cannabis (i.e. ‘skunk’)	NA	69 (90.8%)	NA	59 (83.1%)	
Cannabis, amount used on a day of use (grams) (users)	NA	1.1 (0.8) [1.0, 0.1–4.0]	NA	0.6 (0.7) [0.4, 0.03–3.5, *n* = 68]	Adolescent users > adult users*

Group differences are highlighted in the final column. Amount of cannabis used on a day of use has data missing for three adult users. For continuous data mean (SD) [median, minimum–maximum] is shown, for categorical data *n* (%) is shown.

CUDIT-R: cannabis use disorder identification test revised; NA: not applicable; SD: standard deviation.

* *p* < 0.05, **p < 0.01, ***p < 0.001.

### Regressions

Descriptive statistics for outcome variables are presented in Table 3.

**Table 3.** Descriptive statistics for the four outcome variables.

	Adolescent control (*n* = 63)	Adolescent user (*n* = 76)	Adult control (*n* = 64)	Adult user (*n* = 71)	Group differences
BDI	9.90 (7.13) [10.00, 0.00–33.00]	12.71 (8.34) [11.00, 1.00–41.00]	7.30 (7.04) [5.00, 0.00–28.00]	7.94 (8.06) [6.00, 0.00–31.00]	Adolescents > adults***
BAI	11.37 (8.93) [10.00, 0.00–39.00]	13.13 (10.34) [10.50, 0.00–46.00]	7.86 (5.54) [8.00, 0.00–24.00]	7.54 (7.45) [5.00, 0.00–32.00]	Adolescents > adults***
PSI-a	23.05 (14.30) [21.00, 1.00–67.00]	32.71 (20.93) [29.00, 2.00–97.00]	18.53 (15.40) [16.00, 0.00–64.00]	23.07 (17.04) [18.00, 2.00–78.00]	Adolescents > adults** and Users > controls**
Severe DSM-5 CUD					
No		38 (50.0%)		58 (81.7%)	
Yes		38 (50.0%)		13 (18.3%)	Adolescent user > adult user***

Mean (SD) [median, minimum–maximum] are shown.

BAI: Beck anxiety inventory; BDI: Beck depression inventory; DSM-5 CUD: Diagnostic and statistical manual of mental disorders-5 cannabis use disorder; PSI-a: psychotomimetic states inventory-adapted; SD: standard deviation.

** *p* < 0.01, ***p < 0.001.

#### Severe CUD (Figure 1, Table 4 and Supplemental Table S6)

Within users, adolescent-status predicted likelihood of having severe CUD (OR = 4.462, *p* < 0.001, 95% confidence interval (CI): 2.106–9.454) (Table 4). This effect persisted after adjusting for covariates (OR = 3.474, *p* = 0.004, 95% CI: 1.501–8.036).

**Figure 1.**
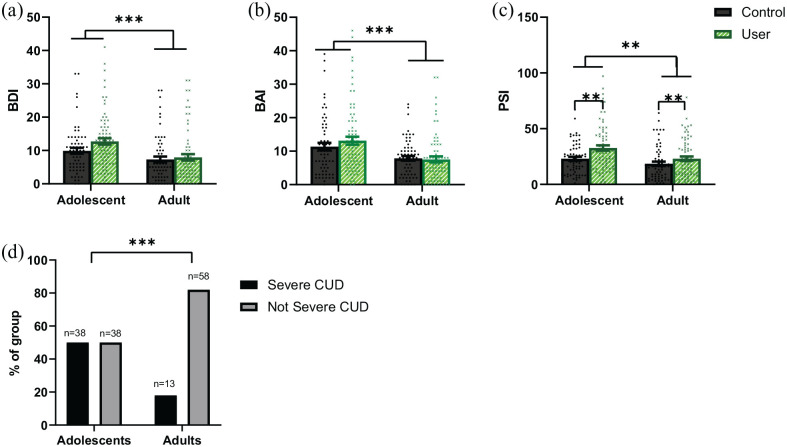
Bar charts showing means, error bars representing 95% CIs around the mean, and distribution of the data in adolescent controls (*n* = 63) and users (*n* = 76), and adult controls (*n* = 64) and users (*n* = 71) for (a) BDI scores. Adolescents have greater BDI scores than adults (***p < 0.001 adjusted and unadjusted), no difference between users and controls, and no interaction. (b) BAI scores. Adolescents have greater BAI scores than adults (***p < 0.001 adjusted and unadjusted), no difference between users and controls, and no interaction. (c) PSI-a scores. Adolescents have greater PSI-a scores than adults (**p = 0.001 unadjusted, *p* = 0.015 adjusted), and users have greater PSI-a scores than controls (**p = 0.01 unadjusted, *p* = 0.014 adjusted). (d) Percentage of adolescent and adult users with and without severe CUD. Adolescents were more likely to have severe CUD than adults (***p < 0.001 unadjusted, *p* = 0.004 adjusted). In (a–c), controls are shown by black circles and dark grey bars; users are shown by green/grey crosses and light green/grey bars with diagonal stripes. In (d), the percentage of participants with severe CUD is shown by the black bar and the percentage of participants without severe CUD is shown by the grey bar. BAI: Beck anxiety inventory; BDI: Beck depression inventory; CI: confidence interval; CUD: cannabis use disorder; PSI-a: psychotomimetic states inventory-adapted

**Table 4.** Summary of regression results.

	Severe CUD (*n* = 147)	Depression (*n* = 274)	Anxiety (*n* = 274)	Psychotic-like symptoms (*n* = 273)
User-group	NA	No	No	**Yes (*b*** **=** **7.121, *p*** **=** **0.001)**
Age-group	**Yes (OR** **=** **4.462, *p*** **<** **0.001)**	**Yes (*b*** **=** **3.766, *p*** **<** **0.001)**	**Yes (*b*** **=** **4.627, *p*** **<** **0.011)**	**Yes (*b*** **=** **3.130, *p*** **=** **0.001)**
User-group X age-group	NA	No	No	No
Adjusted user-group	NA	No	No	**Yes (*b*** **=** **6.004, *p*** **=** **0.014)**
Adjusted age-group	**Yes (OR** **=** **3.474, *p*** **=** **0.004)**	**Yes (*b*** **=** **3.915, *p*** **<** **0.001)**	**Yes (*b*** **=** **4.528, *p*** **<** **0.001)**	**Yes (*b*** **=** **5.509, *p*** **=** **0.015)**

Do the exposure variables (user-group and age-group, and their interaction) significantly predict the outcome variables? Severe CUD models were run only in users (*n* = 147, adjusted models *n* = 143). Bold text highlights if the user-group or age-group main effects or interaction were significant for the four outcome variables.

Depression and anxiety *n* = 274, adjusted models *n* = 268. Psychotic-like symptoms *n* = 273, adjusted model *n* = 267. Adjusted terms are from models including pre-defined covariates: gender, SES, RT-18, daily smoking, AUDIT and other drug use. The best models never included the interaction term; hence, there are no adjusted interaction terms.

AUDIT: alcohol use disorders identification test; *b*: unstandardised beta; CUD: cannabis use disorder; NA: not applicable; OR: odds ratio; RT-18: Risk-Taking 18.

#### Depression (Figure 1, Table 4 and Supplemental Table S7)

Adolescent-status predicted greater BDI score (*b* = 3.766, *p* < 0.001, 95% CI: 1.930–5.601) (Table 4). This effect persisted after adjusting for covariates (*b* = 3.915, *p* < 0.001, 95% CI: 1.994–5.836). User-group and the user-group by age-group interaction, were not significantly related to BDI score.

Bayesian analyses did not support the null hypothesis that users and controls had similar BDI scores (BF_01_ = 1.846) nor did they support the null hypothesis that adolescent users and adolescent controls had similar BDI scores (BF_01_ = 0.935).

#### Anxiety (Figure 1, Table 4 and Supplemental Table S8)

Adolescent-status predicted greater BAI (*b* = 4.627, *p* < 0.001, 95% CI: 2.642–6.612) (Table 4). This effect persisted after adjusting for covariates (*b* = 4.528, *p* < 0.001, 95% CI: 2.384–6.671). User-group and the user-group by age-group interaction were not significantly related to BAI.

Bayesian analyses supported the null hypothesis that users and controls had similar BAI scores (BF_01_ = 7.724) and the null hypothesis that adolescent users and adolescent controls had similar BAI scores (BF_01_ = 4.401).

#### Psychotic-like symptoms (Figure 1, Table 4 and Supplemental Table S9)

User-status predicted greater PSI-a (*b* = 7.121, *p* = 0.001, 95% CI: 3.033–11.465). Adolescent-status predicted greater PSI-a (*b* = 7.254, *p* = 0.001, 95% CI: 3.130–11.378) (Table 4).These effects both persisted after adjusting for covariates (user-group: *b* = 6.004, *p* = 0.014, 95% CI: 1.211–10.796; age-group: *b* = 5.509, *p* = 0.015, 95% CI: 1.070–9.947). The user-group by age-group interaction was not significantly related to PSI-a.

#### Exploratory results (see Supplemental Materials)

The patterns of our results described above were mirrored when considering the relationships between age-group, user-group and clinical categorical outcomes of ‘at least mild depression’ and ‘at least mild anxiety’. Adolescent status increased the risk (depression OR = 2.25; anxiety OR = 1.70), but user-status did not.

After splitting the user-groups into those with and without severe CUD and comparing (1) adult controls (2) adolescent controls, (3) adult users without severe CUD, (4) adolescent users without severe CUD, (5) adult users with severe CUD and (6) and adolescent users with CUD, there were strong linear effects of group in our outcome variables (*p*s < 0.001). Qualitatively, adolescent users with severe CUD had the highest BDI, BAI and PSI-a mean scores (Figures S1, S2 and S3).

When conducting a 2 × 2 (age-group × CUD-status) analysis of variance only in users, there were significant main effects of age-group on BDI (*F*(1,143) = 4.165, *p* = 0.043, $\eta_{p}^{2}=0.028$ ), BAI (*F*(1,143) = 4.299, *p* = 0.040, $\eta_{p}^{2}=0.029$ ) and PSI-a (*F*(1,142) = 4.273, *p* = 0.041, $\eta_{p}^{2}=0.029$ ) and significant main effects of CUD status on BDI (*F*(1,143) = 11.236, *p* = 0.001, $\eta_{p}^{2}=0.073$ ), BAI (*F*(1,143) = 9.815, *p* = 0.002, $\eta_{p}^{2}=0.064$ ) and PSI-a (*F*(1,142) = 4.525, *p* = 0.035, $\eta_{p}^{2}=0.031$ ). However, all interactions were non-significant. Thus, being an adolescent and having severe CUD were additively associated with greater BDI, BAI and PSI-a. After adjusting for our pre-defined covariates and cannabis use frequency, the CUD-status main effect remained for BDI (*F*(1,132) = 9.382, *p* = 0.003, $\eta_{p}^{2}=0.066$ ) and BAI (*F*(1,132) = 7.414, *p* = 0.007, $\eta_{p}^{2}=0.053$ ), but the user-group effect for PSI-a and the age-group effects for all outcomes became non-significant.

## Discussion

This cross-sectional study compared the presence of severe CUD and the severity of mental health symptoms in adolescent and adult cannabis users with gender- and age-matched controls. Adolescent users were significantly more likely to have severe CUD than adult users. Both cannabis user-status and adolescent-status were associated with greater psychotic-like symptoms, additively resulting in adolescent cannabis users having the greatest psychotic-like symptoms. User-status was not associated with subclinical depression or anxiety levels and there was Bayesian support for users and controls having similar anxiety levels. No significant interactions were found between user-group and age-group for subclinical depression, anxiety or psychotic-like symptoms, suggesting that adolescents do not have a greater vulnerability to the associations between chronic cannabis use and mental health problems in comparison to adults. However, our exploratory analyses suggested that severe CUD predicted worse mental health symptoms, which resulted in adolescent users with severe CUD having the highest levels of depression, anxiety and psychotic-like symptoms.

After adjustment for covariates, adolescents had a 3.5 times greater odds of having severe CUD than adults, with 50% of this group endorsing six or more CUD symptoms. This effect size is similar to previous estimates of increased risk (Chen et al., 2009; Ehlers et al., 2010; Le Strat et al., 2015; Wittchen et al., 2011), demonstrating the effect’s replicability. CUD risk was greater in adolescents despite their shorter duration of cannabis use compared to adults. This is notable because previous studies have mainly tested associations between age-of-onset and addiction (and other outcome variables) in adults, where early age-of-onset is also associated with greater duration of cannabis use. Moreover, unlike many previous studies, our adolescent and adult user-groups were matched on cannabis frequency, which therefore excludes this difference as a possible explanation. Furthermore, a similar proportion of adolescents (91%) and adults (83%) used strong herbal cannabis as their usual type, and the delta-9-tetrahydrocannabinol (THC) concentration in both groups’ strong herbal cannabis was the same (21%) (see Supplementary Materials). However, adolescent users reported using more cannabis per day of use (1.1 g) than adults (0.6 g), which may partially contribute to cannabis problems (Callaghan et al., 2020; Tomko et al., 2018; Zeisser et al., 2012). On the other hand, this estimate may be inaccurate (Hindocha et al., 2017, 2018), especially because UK cannabis users buy their cannabis illegally, so the weight purchased may not be known, and estimating the quantity put into a joint (or other method) is difficult. Further work carefully examining relationships between precise cannabis and cannabinoid quantities, or better still, standard THC units (Freeman and Lorenzetti, 2020) and addiction is needed.

We speculate that adolescents may be more sensitive to the development of CUD than adults for a number of reasons, including greater disruption of interpersonal relationships, for example, with parents or teachers; a hyper plastic brain and a developing endocannabinoid system (Meyer et al., 2018); a more malleable social life and evolving sense of identity which can quickly shift towards cannabis use (Hammersley et al., 2001); potentially subtle differences in acute effects of cannabis (Mokrysz et al., 2016; Murray et al., 2022); a greater desire to binge on cannabis (Borissova et al., 2022); and a drive towards social attunement (Cousijn et al., 2018). However, research into the different profiles of adolescent and adult CUD, and the neuropsychopharmacological predictors of CUD onset in adolescents is needed.

We found null relationships between cannabis user-status and subclinical depression and anxiety levels, and no evidence of adolescent vulnerability. Furthermore, the absence of associations between cannabis use frequency and our measures of depression and anxiety in users (see Supplemental Materials) casts further doubt on the impact of cannabis use on levels of anxiety and depression. We also found null relationships between user-status and the presence of clinically relevant anxiety or depression. Previous research has suggested that cannabis use is associated with an augmented risk of depression and anxiety in adults (Crippa et al., 2009; Degenhardt et al., 2001, 2003; Guttmannova et al., 2017; Horwood et al., 2012; Lev-Ran et al., 2014) and associated with greater risks later in life for adolescents (Gobbi et al., 2019). Given that the effect sizes of these relationships from meta-analyses are small (Chen et al., 2010) (OR = 1.17–1.62 for depression and 1.25 for anxiety), and base rate of clinical anxiety or depression is not high, our sample may have been underpowered to detect differences in clinical anxiety and depression.

Nevertheless, it is notable that in our sample of relatively frequent cannabis users, using at a mean frequency of 4 days/week, there was no significant evidence of greater subclinical anxiety or depression levels in cannabis users aged 16–17 years compared to gender- and age-matched controls or cannabis-matched adults. Our study was powered to detect interactions with Cohen’s *f* ⩾ 0.17. For anxiety, the null differences were supported by a Bayesian analysis. There has been inconsistent evidence of heightened cannabis-related vulnerability at younger ages for both disorders (Guttmannova et al., 2017; Hayatbakhsh et al., 2007; Horwood et al., 2012; Hosseini and Oremus, 2019; Leadbeater et al., 2019; Lev-Ran et al., 2014). In line with previous research, our study further suggests there is not yet sufficient evidence to claim that cannabis use during adolescence is associated with a greater risk of higher levels of depression or anxiety compared to cannabis use in adulthood. We should await further longitudinal analyses and studies with clinical diagnoses to corroborate these findings.

Previous research has consistently implicated cannabis in the development of clinical psychosis, psychotic-like, schizotypal and subclinical symptoms (Arseneault et al., 2002; Henquet et al., 2005; Hides et al., 2009; Kuepper et al., 2011; Marconi et al., 2016; Miettunen et al., 2008; Moore et al., 2007), including psychotic-like symptoms during adolescence (Bourque et al., 2018). Likewise, cannabis use was significantly associated with psychotic-like symptoms in our sample of adolescents and adults, a relationship that remained significant after adjusting for covariates. Although we did not measure clinical psychotic disorders, these findings indicate an important augmented risk, given the amplified chance of transitioning to psychosis with subclinical symptoms (Kaymaz et al., 2012; Kuepper et al., 2011). Adolescents overall had greater psychotic-like symptoms than adults; hence, there was an additive effect, resulting in adolescent users showing the greatest severity. Indeed, adolescent users’ mean PSI-a score was 75% higher than that of adult controls. However, age-group did not moderate the impact of user-group. Thus, there was no evidence of synergistic vulnerability.

Our exploratory analyses suggested that there was a consistent pattern in depression, anxiety and psychotic-like symptoms for those with and without severe CUD. Adolescent users with CUD consistently had the highest mean across these three outcomes. Only adolescent users with severe CUD significantly differed from other groups and only they had greater odds of having at least mild anxiety or depression relative to the reference category, adult controls. When analysing users, adolescence and severe CUD were additively and significantly associated with mental health symptoms, explaining why adolescent users with severe CUD have the highest means. However, there were no significant interactions, so severe CUD did not have a *greater* effect in adolescents than adults. Previous research has shown that dependent use of cannabis is particularly strongly associated with mental health problems (Braidwood et al., 2018; van der Pol et al., 2013), while non-dependent frequent use may not be (Braidwood et al., 2018). Our exploratory findings add to these by demonstrating the relevance of CUD to mental health problems in adolescence, despite the fact that user-group differences were absent for anxiety and depression. This is important by virtue of adolescents’ heightened risk of developing CUD and their greater likelihood of existing mental health problems. However, these results should be interpreted cautiously as they were exploratory, the sub-group sample sizes were small (see Supplemental Table S11), adjustment for covariates removed the significant effect of CUD-status on psychotic-like symptoms, and the study was not designed to compare users with and without CUD.

### Strengths and limitations

Direct comparisons of adolescent and adult cannabis users are rare. One major strength of this study is its novel design in which four groups were compared: adolescent and adult cannabis users and age- and gender-matched controls. Crucially, our adolescent and adult users were matched on cannabis use frequency and the proportions of each group who typically use strong herbal cannabis (i.e. skunk) were similar. Controls had been exposed to limited cannabis or tobacco use, reducing unmeasured confounding differences with users. Adult cannabis users had never used cannabis frequently before the age of 18 years, ensuring adolescent development was not substantially impacted by cannabis. Recent abstinence from alcohol and other drugs was biochemically verified. Furthermore, we pre-registered our protocol and analyses and adjusted for pre-defined covariates.

Due to the sampling methodology, the results cannot be interpreted as representative of the general population. However, this approach was necessary to target frequent cannabis users and select matched controls and maximise statistical power to test our hypotheses. This approach is common in observational cannabis research (Morgan et al., 2012; van der Pol et al., 2013), where baseline levels of illicit drug use are relatively low in the general population. Another limitation is the cross-sectional nature of the analysis. As some previous studies imply that there may be a time-lagged effect of cannabis on mental health (Gobbi et al., 2019), this could contribute to our null findings. A further limitation is that we did not conduct DSM clinical interviews for diagnoses of mental disorders; larger epidemiological studies are needed to probe these relationships.

Inevitably, our adolescent users began using cannabis earlier than our adult users. Although we aimed to recruit similar adolescent and adult users via matched gender, age and cannabis frequency, compared demographics and adjusted for relevant covariates, it is still possible that people who initiate cannabis use early in life are qualitatively different from those who initiate cannabis use later in life in ways we did not account for. These pre-existing differences could have impacted our results. Crucially, however, age-of-onset in adult users was not associated with mental health symptomatology and the extant literature concerning these relationships is conspicuously mixed (Guttmannova et al., 2017; Lev-Ran et al., 2014). Moreover, arguably, we cannot disaggregate current age, age-of-onset and duration of use. Triangulation of existing and future longitudinal research will allow conclusions about the impacts of these specific, closely related exposure variables to be drawn. We have therefore followed up our participants on four further testing occasions every 3 months over 1 year to provide a snapshot of a developmental profile; these results will be reported in the future.

## Conclusions

In sum, 16- to 17-year olds were not at an interactively greater risk of cannabis-related mental health problems, compared to 26- to 29-year olds. This suggests that adolescents might not be more vulnerable to cannabis harms than adults. More longitudinal research is needed to further test this suggestion. However, adolescents have an amplified risk of severe CUD relative to adults, which in combination with being young, augments symptoms of mental ill health. Cannabis harm reduction campaigns should therefore highlight the greater risk of addiction to cannabis during adolescence.

## Supplemental Material

sj-docx-1-jop-10.1177_02698811221108956 – Supplemental material for The CannTeen Study: Cannabis use disorder, depression, anxiety, and psychotic-like symptoms in adolescent and adult cannabis users and age-matched controls Supplemental material, sj-docx-1-jop-10.1177_02698811221108956 for The CannTeen Study: Cannabis use disorder, depression, anxiety, and psychotic-like symptoms in adolescent and adult cannabis users and age-matched controls by Will Lawn, Claire Mokrysz, Rachel Lees, Katie Trinci, Kat Petrilli, Martine Skumlien, Anna Borissova, Shelan Ofori, Catherine Bird, Grace Jones, Michael AP Bloomfield, Ravi K Das, Matthew B Wall, Tom P Freeman and H Valerie Curran in Journal of Psychopharmacology
