# Supplementary material for: The CannTeen Study: Cannabis use disorder, depression, anxiety, and psychotic-like symptoms in adolescent and adult cannabis users and age-matched controls
Source: J Psychopharmacol. 2022 Jun 30;36(12):1350–61. doi: 10.1177/02698811221108956 (PMC9716489; doi:10.1177/02698811221108956)
Supplement: sj-docx-1-jop-10.1177_02698811221108956 – Supplemental material for The CannTeen Study: Cannabis use disorder, depression, anxiety, and psychotic-like symptoms in adolescent and adult cannabis users and age-matched controls [file sj-docx-1-jop-10.1177_02698811221108956.docx]

**CannTeen: Cannabis use disorder, depression, anxiety, and psychotic-like symptoms in adolescent and adult cannabis users and age-matched controls**

**Supplementary Materials**

**Authors**

Will Lawn^1,2,3^, Claire Mokrysz^3^, Rachel Lees^4^, Katie Trinci^3^, Kat Petrilli^4^, Martine Skumlien^5^, Anna Borissova^6,7^, Shelan Ofori^3^, Catherine Bird^8^, Grace Jones^3^, Michael AP Bloomfield^3,7,9^, Ravi K Das^3^, Matthew B Wall^3,10^, Tom P Freeman^3,4^ & H Valerie Curran^3^

**Affiliations**

1 Department of Psychology, Institute of Psychiatry, Psychology and Neuroscience, King’s College London, London, United Kingdom; 2 National Addiction Centre, Institute of Psychiatry, Psychology and Neuroscience, King’s College London, London, United Kingdom; 3 Clinical Psychopharmacology Unit, University College London, London, United Kingdom; 4 Addiction and Mental Health Group (AIM), Psychology Department, University of Bath, Bath, United Kingdom; 5 Department of Psychiatry, University of Cambridge, Cambridge, United Kingdom; 6 Department of Neuroimaging, Institute of Psychiatry, Psychology and Neuroscience, King’s College London, London, United Kingdom; 7 NIHR University College London Hospitals Biomedical Research Centre, University College Hospital, London, United Kingdom.; 8 Centre for Affective Disorders, Institute of Psychiatry, Psychology and Neuroscience, King’s College London, London, United Kingdom; 9 Translational Psychiatry Research Group, Research Department of Mental Health Neuroscience, Division of Psychiatry, University College London, London, United Kingdom; 10 Invicro London, Burlington Danes Building, Hammersmith Hospital, Du Cane Road, London, United Kingdom.

**Table of Contents**

[**Section 1: Background Information** 2](#_Toc103290857)

[**Section 2: Inclusion and Exclusion Criteria** 3](#_Toc103290858)

[**Section 3: Measures and Statistical Analyses** 5](#_Toc103290859)

[**3.1 Other measures** 5](#_Toc103290860)

[**3.2 Pre-defined covariates** 5](#_Toc103290861)

[**3.3 Predictor and outcome variables** 6](#_Toc103290862)

[**3.4 Data Pre-processing** 7](#_Toc103290863)

[**3.5 Linear and Logistic Regression Models & Assumptions** 7](#_Toc103290864)

[**Section 4. Participants** 10](#_Toc103290865)

[**Section 5. Results from Logistic and Linear Regression Models** 13](#_Toc103290866)

[**Section 6. Exploratory results** 18](#_Toc103290867)

[**6.1 Clinical thresholds for BDI and BAI** 18](#_Toc103290868)

[**6.2 Relationship between severe CUD and depression, anxiety and psychotic-like symptoms** 20](#_Toc103290869)

[**6.2.1 BDI** 20](#_Toc103290870)

[**6.2.2 BAI** 22](#_Toc103290871)

[**6.2.3 PSI-a** 24](#_Toc103290872)

[**6.3 Correlations between age-of-onset and mental health symptoms in adult users** 26](#_Toc103290873)

[**6.4 Cannabis type and THC concentration** 26](#_Toc103290874)

[**Section 7. Supplementary Discussion** 27](#_Toc103290875)

# **Section 1: Background Information**

Cannabis is particularly popular in adolescents, with 19.3% of 15 year-olds reported using cannabis in the past year, compared to 13.4% of 25-29 year olds and 7.3% of 30-34 year olds (NHS-Digital, 2018, 2019).

Cannabis is notably more popular amongst 15-year olds than other popular illicit drugs, including nitrous oxide (where 10.9% reported use in the last year), cocaine (4.2%) and ecstasy (3.9%) (NHS-Digital, 2018).

More than 75% of English <18 year-olds receiving treatment for a drug problem receive it primarily for cannabis. The second most common primary problem drug for <18s was alcohol, at 14% (NDTMS, 2019). From 2006 to 2019, there was a 40% increase in the number of young people receiving cannabis treatment, while there were falls of between 37-91% for cocaine, alcohol, amphetamine and opiates. Hence, cannabis is a particularly popular drug for adolescents in the UK and treatment demand for cannabis has increased in the past decade.

# **Section 2: Inclusion and Exclusion Criteria**

**Table S1**. Inclusion and exclusion criteria for all participants, and specific criteria for each group. BMI=Body Mass Index

|  | Inclusion criteria | Exclusion criteria |
| --- | --- | --- |
| All participants | - Able to come to UCL five times over the next year - Capacity to give informed consent - Normal or corrected-to-normal vision - Fluent in English | - Any illicit drug use within 48 hours of the behavioural baseline session, verified with self-report and saliva testing - Any cannabis or alcohol use within 12 hours of the behavioural baseline session, verified with self-report and saliva/breathalyser testing. - Personal history of a diagnosed psychotic episode or disorder - Any one illicit drug taken >2 days/month (averaged over last 3 months) (except laughing gas) - Use of laughing gas >1 day/week (averaged over last 3 months) - Receiving treatment for any mental health condition, including cannabis dependence, in the last month - Unwilling to give blood samples or likely to faint on blood sampling - Current daily use of a medication which is commonly psychotropic - Any mental or physical health problem judged to be problematic for the study, by a medical doctor |
| Teenage cannabis users | - Aged 16-17 years - Cannabis use at a frequency of 1-7 days/week (averaged over last 3 months) | - Age-adjusted BMI <2^nd^ percentile or >99.6^th^ percentile |
| Teenage controls | - Aged 16-17 years - Between 1 and 10 days of lifetime cannabis use ***or*** 0 days of lifetime cannabis use and at least 1 day of lifetime cigarette/roll-up use | - Age-adjusted BMI <2^nd^ percentile or >99.6^th^ percentile - Cannabis use more than once in the last 3 months before behavioural baseline session - Cannabis use in the month prior to the behavioural baseline session |
| Adult cannabis users | - Aged 26-29 years - Cannabis use at a frequency of 1-7 days/week (averaged over last 3 months) | - Before the age of 18, cannabis use at a frequency of once per week or more for a period of 3 months or more. - BMI <18.5 or BMI>34.9 |
| Adult controls | - Aged 26-29 years - Between 1 and 10 days of lifetime cannabis use ***or*** 0 days of lifetime cannabis use and at least 1 day of lifetime cigarette/roll-up use | - Cannabis use more than once in the last 3 months before behavioural baseline session - Cannabis use in the month prior to the baseline session - BMI <18.5 or BMI>34.9 |

# **Section 3: Measures and Statistical Analyses**

## **3.1 Other measures**

**Gender**: We asked participants what their gender was. Participants could respond ‘male’, ‘female’, ‘other’, or ‘prefer not to say’. Participants only responded male or female. Female was coded 0 and male was coded 1.

**Substance use frequency:** Cannabis, alcohol, tobacco and illicit drug use frequency were all assessed using the TLFB method (Robinson et al., 2014). Participants stated which type of cannabis they used. Cannabis was categorized into strong herbal cannabis (i.e. high-strength, ‘skunk’, ‘high-grade’, sinsemilla, seedless, indoor-grown); weak herbal (i.e. low-strength herbal, outdoor-grown seeded cannabis, ‘Thaiweed’, ‘brickweed’, ‘ditchweed’); and hash (i.e. resin, the brown solid created by pressing resin glands/trichomes) (Freeman & Winstock, 2015). Participants were prompted to recall the days on which they used these drugs in the 12 weeks prior to baseline. An average use frequency for each drug was calculated in days/week. For pre-defined covariates, participants were categorized into the following groups: daily cigarette or roll-up tobacco use (≥ 6.5 days/week) (coded 1) or non-daily use (coded 0); and illicit drug use≥1 day/month (coded 1) or illicit drug use<1 day/month (coded 0).

**Delta-9-tetrahydrocannabinol (THC) concentration:** Cannabis users were asked if they were willing to donate a small amount (roughly 0.3g) of their usual cannabis to us. The cannabis was then analysed for THC concentration using ultra high performance liquid chromatography (UPLC).

**Breathalyzer Test:** Participants blood alcohol levels were assessed using a Lion Alcometer 500 breathalyzer at baseline, to check alcohol abstinence. Only those with a blood alcohol of 0 completed the session.

**Saliva drug tests:** Instant saliva drugs tests (either Alere DDSV 703 or ALLTEST DSD-867MET/C) were administered at baseline, assessing delta-9-tetrahydrocannabinol (THC), opiates, benzodiazepine, methamphetamine, amphetamine and opiates in saliva. Only those with negative saliva tests for all drugs completed the session.

**Risk-Taking 18 (RT18):** An 18-item questionnaire assessing risk-taking behaviour. Total scores range from 0-18, with higher scores indicating higher levels of risk taking behaviour (De Haan et al., 2011)

**Socio-economic status (SES):** Assessed using participants’ mother’s educational level. Participants are categorized into those whose mothers have an educational level of undergraduate degree or above (coded 1), and those whose mothers have an educational level below undergraduate degree (coded 0).

**Alcohol Use Disorder Identification Test (AUDIT):** A 10-item questionnaire assessing hazardous drinking and dependence. Scores range from 0-40 (Bohn & Babor, 1995).

**Cannabis Use Disorder Identification Test- Revised (CUDIT-R):** An 8-item questionnaire assessing hazardous cannabis use and dependence. Scores range from 0-32 (Adamson et al., 2010)

**Familial mental health disorders:** we asked participants if any first-degree relative (parent, sibling, child) had been diagnosed with any of the following disorders: psychosis, depression, anxiety, alcohol problems, or illicit drug problems.

## **3.2 Pre-defined covariates**

*Pre-defined covariates summary*:

- Gender. Coded as: 0=female; 1=male
- Mother’s educational level. Coded as 0=below undergraduate degree; 1=undergraduate degree or above
- RT-18 total score
- AUDIT total score
- Daily tobacco use. Coded as 0=non-daily; 1=daily.

Monthly other illicit drug use: Coded as 0=less than monthly; 1=1 or more days per

## **3.3 Predictor and outcome variables**

*Predictor variable*s:

- Age-Group. Coded as: 0=adults; 1=teenagers
- User-Group. Coded as: 0=controls; 1=users
- Frequency of cannabis use, in days-per-week.

*Outcome variables*:

- BDI total score
- BAI total score
- PSI-a total score
- Severe CUD

## **3.4 Data Pre-processing**

Missing questionnaire items were imputed using single-value substitution, where the number of missing items is <30% total items (Gmel, 2001; Hawthorne & Elliott, 2005; Shrive et al., 2006). The missing items were replaced with the overall participant’s mean response for that questionnaire, rounded to the nearest integer. Any questionnaires with >30% missing items were deleted entirely and treated as missing data.

Questionnaire totals were Winsorized, within each group, using Tukey’s method, i.e. totals which were greater than three times the interquartile range away from the upper or lower quartile of the distribution were replaced with the nearest non-outlying datapoint. Impossible answers to any questions were deleted and treated as missing.

Note, this cross-sectional mental health and addiction data had previously been provisionally analysed for a masters dissertation and reported as a poster presentation (Lawn et al., 2020). Our hypotheses remained the same throughout.

**Table S2**. Number of participants who had <30% data missing which was then imputed and number of participants where data was excluded due to number of missing items in a questionnaire >30%.

| Variable | Participants with some missing data, <30% | Participants with fully missing data, >30% |
| --- | --- | --- |
| PSI | 16 | 1 |
| BDI | 4 | 0 |
| BAI | 12 | 0 |
| RT | 5 | 0 |
| AUDIT | 0 | 0 |
| CUDIT | 2 | 0 |
| DSM-5 CUD | 4 | 0 |

**Table S3**. Number of datapoints that were Winsorized using Tukey’s method

| Variable | Number of datapoints |
| --- | --- |
| BDI | 3 |
| BAI | 0 |
| PSI | 0 |

## **3.5 Linear and Logistic Regression Models & Assumptions**

First, we ran a logistic regression model only in users for severe CUD with age-group as the only predictor. Subsequently, we ran linear regression models in all participants for BDI, BAI, and PSI-a, in which user-group was entered in first block, then user-group and age-group in the second block, and their interaction in the third block. Then we added our pre-defined covariates to the best model for each outcome variable. This was always the model with both main effects, but not the interaction.

Additionally, we ran models in only users for BDI, BAI, PSI-a, and severe CUD with cannabis use frequency and age-group in the second block, and their interaction in their third block. Then we added our pre-defined covariates to the best model for each outcome variable. This was always the model with both main effects, but not the interaction. Within users, this tested the relationship between cannabis use frequency and our outcomes, as well as testing whether this relationship was moderated by age-group

**Linear Regression models – assumptions**

With all linear regression models, we checked for multicollinearity of predictors (using correlations and VIF values), normality of residuals (using visual inspection of histograms, Kolmogrov-Smirnov tests, skewness and kurtosis values), cases of influence (using Cook’s distances), heteroscedasticity of variance and independence of errors, to ensure the models met assumptions.

For each linear regression model, we assessed R-square change values and interpreted the predictors’ unstandardised betas and their 95% confidence intervals.

All assumptions were met for all models. Residuals in the models were visually inspected and were normal, homogeneity of variance of residuals was met, Durbin-Watson values were near 2, Cook’s distances were very small (<0.1) and correlations between predictors were acceptably small.

**Logistic Regression models - assumptions**

With all logistic regression models we checked adequate presence of data in the yes/no categories for both groups, multicollinearity of predictors, and cases of influence to ensure the data meet assumptions.

We interpret odds ratios and 95% confidence intervals for the predictors. We consider –2 log likelihood and Nagelkerke R-square values of the models.

**Table S4**. Logistic and linear regression models in all participants. CUD=cannabis use disorder, BDI=Beck Depression Inventory, BAI=Beck Anxiety Inventory, PSI-a=Adapted psychotomimetic states inventory

| Outcome variable | Model | Predictor variables |
| --- | --- | --- |
| Severe CUD | Model 1a | Age-group |
|  | Model 1b | Age-group and covariates |
| BDI | Model 2a | User-group |
|  | Model 2b | User-group and age-group |
|  | Model 2c | User-group, age-group and user-group by age-group interaction |
|  | Model 2d | User-group, age-group (best model) and covariates |
| BAI | Model 3a | User-group |
|  | Model 3b | User-group and age-group |
|  | Model 3c | User-group, age-group and user-group by age-group interaction |
|  | Model 3d | User-group, age-group (best model) and covariates |
| PSI-a | Model 4a | User-group |
|  | Model 4b | User-group and age-group |
|  | Model 4c | User-group, age-group and user-group by age-group interaction |
|  | Model 4d | User-group, age-group (best model) and covariates |

**3.6 Exploratory statistical analyses**

*Clinical cut-offs*

For BDI, a score of 14 or above was considered mild depression or above. For BAI, a score of 8 or above was considered mild anxiety or above.

We conducted logistic regression analyses to determine if user-group, age-group and their interaction predicted presence of mild depression or anxiety.

We did not conduct logistic regressions with PSI-a because this does not have a validated cut-off.

*Correlations*

We conducted Pearson correlations to test the interrelationships between BDI, BAI and PSI-a. We also conducted Pearson correlations to test associations between age-of-onset and BDI, BAI and PSI-a in only adult users.

*Relationships between severe CUD and depression, anxiety, and psychotic-like symptoms*

To test for associations between severe CUD and mental health symptoms, we split the participants into the following groups, with the following codes:

- Adult controls (1)
- Adolescent controls (2)
- Adult users without severe CUD (3)
- Adolescent users without severe CUD (4)
- Adult users with severe CUD (5)
- Adolescent users with severe CUD (6)

First, we ran one-way ANOVAs with a factor of group, with six levels as the groups above, and BDI, BAI and PSI-a as outcome variables. Although this approach loses the ability to detect interactions, it allows the comparison between control groups and user-groups with and without severe CUD. We investigated linear relationships between group and outcome variables. We conducted Bonferroni-corrected t-tests to investigate where differences lay between groups.

Second, we ran logistic regressions to investigate how these groups predicted presence of mild anxiety or depression, with adult controls as the reference category. We did not include PSI-a because this does not have a validated cut-off.

Third, only in users, we ran two-way ANOVAs with factors of age-group and CUD-status (severe CUD or not). This allowed us to statistically separate the impact of age-group and CUD-status and test their interaction, but only within users. Fourth, we included our pre-defined covariates and cannabis use frequency in ANCOVA models, to test if the effects were robust to adjustment, especially given participants with severe CUD have higher frequency of cannabis use.

# **Section 4. Participants**

Significantly more adolescents were in the high SES category than adults (𝜒^2^_1_=5.345, p=0.021), they used alcohol less frequently than adults (F_1,270_=44.114, p<0.001, η_p_^2^=0.140), and they had higher RT-18 scores than adults (F_1,270_=21.077, p<0.001, η_p_^2^=0.072)

Users had higher AUDIT score than controls (F_1,270_=7.099, p=0.008, η_p_^2^=0.026), they were more likely to use tobacco on a daily basis than controls (𝜒^2^_1_=8.467, p=0.004), they had higher RT-18 scores than controls (F_1,270_=14.847, p<0.001, η_p_^2^=0.052), and were more likely to use other illicit drugs on a monthly basis than controls (𝜒^2^_1_=61.103, p<0.001). Additionally, adolescent users were more likely to use other illicit drugs on a monthly basis than adult users (𝜒^2^_1_=17.183, p<0.001).

For controls, adults had used cannabis slightly more times (4.5) in their life than adolescents (3.4) (t_125_=2.067, p=0.041, d=0.367) and a greater proportion of adult controls had ever used cannabis than adolescent controls (𝜒^2^_1_=4.011, p=0.045) (Table 2). For users, adolescents (14.6 years) first tried cannabis at an earlier age than adults (18.5 years) (t_144_=9.647, p<0.001, d=1.598) and they reported using more cannabis (1.1g) on a day of use than adults (0.6g) (t_142_=3.623, p<0.001, d=0.605).

*Number of DSM-5 CUD symptoms*

Adolescent users averaged 5.2 (SD=2.7, range: 0-11) symptoms, while adult users averaged 3.5 (SD=2.3, range: 0-9) symptoms (t_145_=4.121, p<0.001, d=0.678).

*Type and strength of cannabis*

A similar number of adolescent users (n=69, 90.8%) and adult users (n=59, 83.1%) used strong herbal cannabis as their most common type of cannabis, and these distributions did not differ (χ_3_^2^=3.866, p=0.276) (table S12). Furthermore, albeit in a small subsample, the adolescent users (21.1%, SD=5.2, n=14) and adult users (21.3%, SD=4.6, n=12), used strong cannabis of a similar THC concentration (t_24_=0.100, p=0.921, d=0.041).

*First degree relative mental health*

There were no significant associations between the group participants were in and the presence of mental health disorder diagnoses in first-degree relatives. Two-5 people in each group reported a relative with psychosis; 15-18 people in each group reported a relative with depression; 9-15 people in each group reported a relative with anxiety; 3-9 people in each group reported a relative with alcohol problems; and 1-6 people in each group reported a relative with illicit drug problems (all χ^2^<8.5, p>0.2).

**Table S5**. Lifetime drug use variables for adolescent controls, adolescent users, adult controls and adult users for ever (lifetime) use and use within the past 12 weeks: n (%). Use of e-cigarettes in past 12 months is missing for one adolescent user.

|  | Adolescent control | Adolescent user | Adult control | Adult user |
| --- | --- | --- | --- | --- |
| Alcohol: ever used | 61 (96.8%) | 75 (98.7%) | 64 (100.0%) | 71 (100.0%) |
| Alcohol: used in past 12 weeks: | 55 (87.3%) | 71 (93.4%) | 61 (95.3%) | 67 (94.4%) |
| Tobacco (non-cannabis, i.e. cigarettes & roll-ups): ever used | 49 (77.8%) | 74 (97.4%) | 58 (90.6%) | 66 (93.0%) |
| Tobacco (non-cannabis, i.e. cigarettes & roll-ups): used in past 12 weeks: | 18 (28.6%) | 59 (77.6%) | 18 (28.1%) | 39 (54.9%) |
| Ecstasy: ever used | 5 (7.9%) | 54 (71.1%) | 21 (32.8%) | 52 (73.2%) |
| Ecstasy: used in past 12 weeks | 1 (1.6%) | 35 (46.1%) | 2 (3.1%) | 22 (31.0%) |
| Laughing gas: ever used | 22 (34.9%) | 66 (86.8%) | 24 (37.5%) | 43 (60.6%) |
| Laughing gas: used in past 12 weeks | 4 (6.3%) | 37 (48.7%) | 1 (1.6%) | 5 (7.0%) |
| E-cigarette: ever used | 33 (52.4%) | 50 (65.8%) | 16 (25.0%) | 36 (50.7%) |
| E-cigarette: used in past 12 weeks | 10 (15.9%) | 9 (12.0%) | 3 (4.7%) | 9 (12.7%) |
| Ketamine: ever used | 7 (11.1%) | 48 (63.2%) | 10 (15.6%) | 35 (49.3%) |
| Ketamine: used in past 12 weeks | 4 (6.3%) | 36 (47.4%) | 1 (1.6%) | 10 (14.1%) |
| LSD: ever used | 1 (1.6%) | 29 (38.2%) | 3 (4.7%) | 27 (38.0%) |
| LSD: used in past 12 weeks | 0 (0.0%) | 10 (13.2%) | 0 (0.0%) | 5 (7.0%) |
| Cocaine: ever used | 1 (1.6%) | 28 (36.8%) | 18 (28.1%) | 52 (73.2%) |
| Cocaine: used in past 12 weeks | 1 (1.6%) | 13 (17.1%) | 6 (9.4%) | 23 (32.4%) |
| Magic mushrooms: ever used | 1 (1.6%) | 16 (21.1%) | 12 (18.8%) | 39 (54.9%) |
| Magic mushrooms: used in past 12 weeks | 0 (0.0%) | 3 (3.9%) | 3 (4.7%) | 6 (8.5%) |
| Alprazolam ‘Xanax’: ever used | 0 (0.0%) | 23 (42.6%) | 3 (5.0%) | 10 (17.2%) |
| Alprazolam ‘Xanax’: used in past 12 weeks | 0 (0.0%) | 4 (7.5%) | 0 (0.0%) | 2 (3.4%) |
| Diazepam ‘Valium’: ever used | 0 (0.0%) | 13 (24.1%) | 6 (10.0%) | 12 (21.1%) |
| Diazepam ‘Valium’: used in past 12 weeks | 0 (0.0%) | 3 (5.7%) | 1 (1.7%) | 0 (0.0%) |
| Amphetamine: ever used | 1 (1.6%) | 13 (17.1%) | 2 (3.1%) | 21 (29.6%) |
| Amphetamine: used in past 12 weeks | 0 (0.0%) | 5 (6.6%) | 0 (0.0%) | 4 (5.6%) |
| Synthetic cannabinoids: ever used | 0 (0.0%) | 5 (6.6%) | 2 (3.1%) | 11 (15.5%) |
| Synthetic cannabinoids: used in past 12 weeks | 0 (0.0%) | 0 (0.0%) | 0 (0.0%) | 0 (0.0%) |
| Methamphetamine: ever used | 0 (0.0%) | 0 (0.0%) | 1 (1.6%) | 2 (2.8%) |
| Methamphetamine: used in past 12 weeks | 0 (0.0%) | 0 (0.0%) | 0 (0.0%) | 1 (1.4%) |
| Heroin: ever used | 0 (0.0%) | 0 (0.0%) | 1 (1.6%) | 0 (0.0%) |
| Heroin: used in past 12 weeks | 0 (0.0%) | 0 (0.0%) | 0 (0.0%) | 0 (0.0%) |
| Crack cocaine: ever used | 0 (0.0%) | 0 (0.0%) | 0 (0.0%) | 2 (2.8%) |
| Crack cocaine: used in past 12 weeks | 0 (0.0%) | 0 (0.0%) | 0 (0.0%) | 0 (0.0%) |

# **Section 5. Results from Logistic and Linear Regression Models**

**Table S6.** Logistic regression models (model 1a and 1b) with outcome variable severe cannabis use disorder (CUD) and exposure variable age-group, in users.

Model 1a includes age-group as a predictor (adolescents and adult) (n=147). Model 1b (n=143) includes age-group and predefined covariates (gender, socioeconomic status (SES), risk taking (RT-18), daily smoking, alcohol use disorder identification test (AUDIT) and other drug use). CI = confidence interval.

|  | -2 log likelihood | Nagelkerke R^2^ | P for change in model | Predictor | Unstandardised beta | Standard error | Wald | p | Odds ratio | 95% CI odds ratio: lower | 95% CI odds ratio: upper | N |
| --- | --- | --- | --- | --- | --- | --- | --- | --- | --- | --- | --- | --- |
| Model 1a | **172.959** | **0.149** | **<0.001** | **Age-group** | **1.495** | **0.383** | **15.235** | **<0.001** | **4.462** | **2.106** | **9.454** | 147 |
| Model 1b | 164.725 | 0.183 | 0.470 | **Age-group** | **1.245** | **0.428** | **8.465** | **0.004** | **3.474** | **1.501** | **8.036** | 143 |
|  |  |  |  | Gender | 0.090 | 0.388 | 0.053 | 0.817 | 1.094 | 0.511 | 2.342 |  |
|  |  |  |  | SES | -0.442 | 0.391 | 1.279 | 0.258 | 0.643 | 0.299 | 1.383 |  |
|  |  |  |  | RT-18 | 0.071 | 0.058 | 1.536 | 0.215 | 1.074 | 0.959 | 1.202 |  |
|  |  |  |  | Daily Smoker | 0.387 | 0.547 | 0.500 | 0.479 | 1.472 | 0.504 | 4.296 |  |
|  |  |  |  | AUDIT | 0.038 | 0.043 | 0.782 | 0.377 | 1.038 | 0.955 | 1.129 |  |
|  |  |  |  | Other drug use | 0.267 | 0.417 | 0.411 | 0.521 | 1.306 | 0.577 | 2.956 |  |

|  | Change in R^2^ squared | P for change in model | df1, df2 | Predictor | Unstandardised beta | Standard error | 95% CI beta: lower | 95% CI beta: upper | p | N |
| --- | --- | --- | --- | --- | --- | --- | --- | --- | --- | --- |
| Model 2a | 0.013 | 0.059 | 1, 272 | User-Group | 1.818 | 0.960 | -0.073 | 3.708 | 0.059 | 274 |
| Model 2b | **0.056** | **<0.001** | **1, 271** | User-Group | 1.739 | 0.935 | -0.101 | 3.579 | 0.064 | 274 |
|  |  |  |  | **Age-Group** | **3.766** | **0.932** | **1.930** | **5.601** | **<0.001** |  |
| Model 2c | 0.005 | 0.249 | 1, 270 | User-Group | 0.647 | 1.329 | -1.969 | 3.263 | 0.627 | 274 |
|  |  |  |  | Age-Group | 2.608 | 1.368 | -0.086 | 5.301 | 0.058 |  |
|  |  |  |  | User-Group X Age-Group | 2.159 | 1.868 | -1.519 | 5.837 | 0.249 |  |
| Model 2d (from 2b) | **0.068** | **0.003** | **6, 259** | User-Group | 1.360 | 1.055 | -0.717 | 3.437 | 0.198 | 268 |
|  |  |  |  | **Age-Group** | **3.915** | **0.976** | **1.994** | **5.836** | **<0.001** |  |
|  |  |  |  | **Gender** | **-2.227** | **0.910** | **-4.019** | **-0.435** | **0.015** |  |
|  |  |  |  | **SES** | **-2.606** | **0.918** | **-4.414** | **-0.797** | **0.005** |  |
|  |  |  |  | **RT-18** | **0.260** | **0.125** | **0.013** | **0.507** | **0.039** |  |
|  |  |  |  | Daily Smoker | -0.353 | 1.648 | -3.598 | 2.891 | 0.830 |  |
|  |  |  |  | AUDIT | 0.062 | 0.112 | -0.158 | 0.282 | 0.579 |  |
|  |  |  |  | Other drug use | 0.090 | 1.273 | -2.416 | 2.597 | 0.943 |  |

**Table S7.** Linear regression models (2a, 2b and 2d) with outcome variable Beck Depression Inventory (BDI) and exposure variables user-group and age-group in all participants.

Model 2a includes user-group only (cannabis user and controls) (n=274); Model 2b includes user-group and age-group (adults and adolescents) (n=274). Model 2c includes user-group, age-group and their interaction (n=274). Model 2d (n=268) is an adjusted version of model 2b which includes covariates (gender, socioeconomic status (SES), risk taking (RT-18), daily smoker, alcohol use disorder identification test (AUDIT) score and other drug use) (n=268, due to missing data). CI=confidence interval.

**Table S8**. Linear regression models (models 3a, 3b, 3c and 3d) with outcome variable Beck Anxiety Inventory (BAI) and exposure variables user-group and age-group, in all participants.

Model 3a includes user-group only (n=274); model 3b includes user-group and age-group as predictors (n=274); model 3c includes user-group, age-group and their interaction (n=274); and model 3d (n=268) is an adjusted version of 3b in which the covariates are also included (gender, socioeconomic status (SES), risk taking (RT-18), daily smoker, alcohol use disorder identification test (AUDIT) score and other drug use). CI=confidence interval.

|  | Change in R^2^ squared | P for change in model | df1, df2 | Predictor | Unstandardised beta | Standard error | 95% CI beta: lower | 95% CI beta: upper | p | N |
| --- | --- | --- | --- | --- | --- | --- | --- | --- | --- | --- |
| Model 3a | 0.002 | 0.429 | 1, 272 | User-Group | 0.830 | 0.048 | -1.231 | 2.892 | 0.429 | 274 |
| Model 3b | **0.072** | **<0.001** | **1, 271** | User-Group | 0.733 | 0.042 | -1.257 | 2.723 | 0.469 | 274 |
|  |  |  |  | **Age-Group** | **4.627** | **0.268** | **2.642** | **6.612** | **<0.001** |  |
| Model 3c | 0.004 | 0.302 | 1, 270 | User-Group | -0.324 | 1.438 | -3.154 | 2.506 | 0.822 | 274 |
|  |  |  |  | **Age-Group** | **3.506** | **1.480** | **0.592** | **6.420** | **0.019** |  |
|  |  |  |  | User-Group X Age-Group | 2.091 | 2.021 | -1.889 | 6.070 | 0.302 |  |
| Model 3d (from 3b) | **0.050** | **0.023** | **6, 259** | User-Group | 0.862 | 1.177 | -1.456 | 3.179 | 0.465 | 268 |
|  |  |  |  | **Age-Group** | **4.528** | **1.089** | **2.384** | **6.671** | **<0.001** |  |
|  |  |  |  | **Gender** | **-3.273** | **1.015** | **-5.272** | **-1.273** | **0.001** |  |
|  |  |  |  | SES | -1.138 | 1.025 | -3.156 | 0.880 | 0.268 |  |
|  |  |  |  | RT-18 | 0.172 | 0.140 | -0.103 | 0.448 | 0.220 |  |
|  |  |  |  | Daily Smoker | -1.280 | 1.839 | -4.901 | 2.341 | 0.487 |  |
|  |  |  |  | AUDIT | 0.118 | 0.125 | -0.127 | 0.364 | 0.343 |  |
|  |  |  |  | Other drug use | -0.340 | 1.420 | -3.137 | 2.457 | 0.811 |  |

**Table S9**. Linear regression models (4a, 4b and 4d) with outcome variable Psychotomimetic States Inventory-adapted (PSI-a) and exposure variables user-group and age-group in all participants.

Model 4a includes user-group only (n=273); model 4b includes user-group and age-group as predictors (n=273); model 4c includes user-group, age-group and their interaction (n=273); and model 3d (n=267) is an adjusted version of 3b in which the following covariates were included (gender, socioeconomic status (SES), risk taking (RT-18), daily smoker, alcohol use disorder identification test (AUDIT) score and other drug use).  CI = confidence interval.

|  | Change in R^2^ squared | P for change in model | df1, df2 | Predictor | Unstandardised beta | Standard error | 95% CI beta: lower | 95% CI beta: upper | p | N |
| --- | --- | --- | --- | --- | --- | --- | --- | --- | --- | --- |
| Model 4a | **0.041** | **0.001** | **1, 271** | **User-Group** | **7.249** | **2.141** | **3.033** | **11.465** | **0.001** | 273 |
| Model 4b | **0.041** | **0.001** | **1, 270** | **User-Group** | **7.121** | **2.100** | **2.987** | **11.255** | **0.001** | 273 |
|  |  |  |  | **Age-Group** | **7.254** | **2.095** | **3.130** | **11.378** | **0.001** |  |
| Model 4c | 0.005 | 0.223 | 1, 269 | User-Group | 4.539 | 2.979 | -1.327 | 10.405 | 0.129 | 273 |
|  |  |  |  | Age-Group | 4.516 | 3.068 | -1.524 | 10.556 | 0.142 |  |
|  |  |  |  | User-Group X Age-Group | 5.120 | 4.196 | -3.141 | 13.380 | 0.223 |  |
| Model 4d (from 4b) | **0.060** | **0.007** | **6, 258** | **User-Group** | **6.004** | **2.434** | **1.211** | **10.796** | **0.014** | 267 |
|  |  |  |  | **Age-Group** | **5.509** | **2.254** | **1.070** | **9.947** | **0.015** |  |
|  |  |  |  | Gender | -3.996 | 2.095 | -8.122 | 0.130 | 0.058 |  |
|  |  |  |  | SES | -3.040 | 2.117 | -7.209 | 1.129 | 0.152 |  |
|  |  |  |  | **RT-18** | **0.967** | **0.288** | **0.399** | **1.535** | **0.001** |  |
|  |  |  |  | Daily Smoker | 0.231 | 3.793 | -7.238 | 7.701 | 0.951 |  |
|  |  |  |  | AUDIT | -0.233 | 0.259 | -0.742 | 0.276 | 0.369 |  |
|  |  |  |  | Other drug use | 1.035 | 2.940 | -4.755 | 6.824 | 0.725 |  |

**5.1 Cannabis frequency results**

In users, cannabis use frequency predicted likelihood of having severe CUD (OR=1.401, p=0.001, 95% CI: 1.158–1.695). This effect persisted after including age-group and the pre-defined covariates. However, the interaction between cannabis use frequency and age-group on likelihood of severe CUD was not significant.

Within users, cannabis use frequency did not significantly predict BDI, nor was there a significant interaction between cannabis use frequency and age-group on BDI. Within users, cannabis use frequency did not significantly predict BAI, nor was there a significant interaction between cannabis use frequency and age-group. Within users, cannabis use frequency did not significantly predict PSI-a, nor was there a significant interaction between cannabis use frequency and age-group on PSI-a.

# **Section 6. Exploratory results**

## **6.1 Clinical thresholds for BDI and BAI**

In line with the continuous data analyses in the main text, being an adolescent increased risk of at least mild depression relative to being an adult (OR=2.250, p=0.006, 95% CI: 1.265-4.000), while user-group had no significant relationship with risk of at least mild depression. There was no significant interaction between user-group and age-group.

Being an adolescent increased risk of at least mild anxiety relative to being an adult (OR=1.700, p=0.030, 95% CI: 1.053-1.391), while user-group had no significant relationship with risk of at least mild anxiety. There was no significant interaction between user-group and age-group.

**Table S10.** The number of participants (%) in each group with or without at least mild depression or anxiety. Mild depression is 14 or above on the BDI. Mild anxiety is 8 or above on the BAI.

|  |  | Adolescent control | Adolescent user | Adult control | Adult user |
| --- | --- | --- | --- | --- | --- |
| At least mild depression: |  |  |  |  |  |
|  | No | 48 (76.2%) | 47 (61.8%) | 53 (82.8%) | 59 (83.1%) |
|  | Yes | 15 (23.8%) | 29 (38.2%) | 11 (17.2%) | 12 (17.9%) |
|  |  |  |  |  |  |
| At least mild anxiety: |  |  |  |  |  |
|  | No | 27 (42.9%) | 29 (38.2%) | 30 (46.9%) | 42 (59.2%) |
|  | Yes | 36 (57.1%) | 47(61.8%) | 34 (53.1%) | 29 (40.8%) |

## **6.2 Relationship between severe CUD and depression, anxiety and psychotic-like symptoms**

**Table S11**. Number of people in each CUD sub-group

|  | Adult control | Adolescent control | Adult user without severe CUD | Adult user with severe CUD | Adolescent user without severe CUD | Adolescent user with severe CUD |
| --- | --- | --- | --- | --- | --- | --- |
| n | 64 | 63 | 58 | 13 | 38 | 38 |

### **6.2.1 BDI**

There was a main effect of group (F(5, 268)=7.127, p<0.001, η^2^_p_=0.117), with a strong linear relationship between group and BDI (contrast estimate=5.937, p<0.001).

After Bonferroni-correction for multiple comparisons, only adolescent users with severe CUD differed from any other group; they had significantly greater BDI scores than:

- adult users without severe CUD (t(94)=5.204, p<0.001, mean difference=8.201)
- adolescent controls (t(99)=3.404, p=0.012, mean difference=5.279)
- adult controls (t(100)=5.098, p<0.001, mean difference=7.887)
- and, marginally greater BDI scores than adolescent users without severe CUD (t(74)=2.855, p=0.070, mean difference = 4.947)

Qualitatively, the groups with the lowest BDI means were adult controls and adult users without severe CUD.

In terms of reaching mild depression, only being an adolescent user with severe CUD significantly increased one’s odds relative to the reference category of adult controls (OR=4.818, 95% CI: 1.942-11.957).

When a 2x2 ANOVA was conducted only within users, there were main effects of age-group (F(1,143)=4.165, p=0.043, η^2^_p_=0.028) and CUD-status (F(1,143)=11.236, p=0.001, η^2^_p_=0.073), but no interaction between age-group and CUD-status (F(1,143)=0.010, p=0.921, η^2^_p_<0.001). The main effect of CUD-status (F(1,132)=9.382, p=0.003, η^2^_p_=0.066), but not the main effect of age-group, was robust to the inclusion of covariates, including cannabis use frequency.

**Figure S1.** Beck Depression Inventory (BDI) mean scores, with distributions of data and 95% CIs for adult controls, adolescent controls, adult users without severe CUD, adolescent users without severe CUD (sCUD), adult users with severe CUD and adolescent users with severe CUD. Post-hoc comparisons are Bonferroni-corrected. ***p<0.001, *p<0.05, #p<0.1

### **6.2.2 BAI**

There was a main effect of group (F(5, 268)=7.000, p<0.001, η^2^_p_=0.116), with a strong linear relationship between group and BDI (contrast estimate=5.479, p<0.001).

After Bonferroni-correction for multiple comparisons, adolescent users with severe CUD had greater BAI scores than:

- adult users without severe CUD (t(94)=5.146, p<0.001, mean difference=8.804)
- adult controls (t(100)=4.331, p<0.001, mean difference=7.272)

Also, adolescent users without severe CUD had marginally greater BAI scores than adults without severe CUD (t(94)=2.808, p=0.080, mean difference=4.804). And adolescent controls had greater BAI scores than adult users without severe CUD (t(120)=3.376, p=0.013, mean difference=5.037).

Qualitatively, the groups with the lowest BAI means were adult controls and adult users without severe CUD.

In terms of reaching mild anxiety, only being an adolescent user with severe CUD significantly increased one’s odds relative to the reference category of adult controls (OR=2.843, 95% CI: 1.162-6.955).

When a 2x2 ANOVA was conducted only within users, there were main effects of age-group (F(1,143)=4.299, p=0.040, η^2^_p_ =0.029) and CUD-status (F(1,143)=9.815, p=0.002, η^2^_p_=0.064), but no interaction between age-group and CUD-status (F(1,143)=0.589, p=0.444, η^2^_p_=0.004). The main effect of CUD-status (F(1,132)=7.414, p=0.007, η2p =0.053), but not the main effect of age-group, was robust to the inclusion of covariates, including cannabis use frequency.

**Figure S2.** Beck Anxiety Inventory (BDI) mean scores, with distributions of data and 95% CIs for adult controls, adolescent controls, adult users without severe CUD, adolescent users without severe CUD (sCUD), adult users with severe CUD and adolescent users with severe CUD. Post-hoc comparisons are Bonferroni-corrected. ***p<0.001, *p<0.05, #p<0.1

### **6.2.3 PSI-a**

There was a main effect of group (F(5, 267)=6.454, p<0.001, η^2^_p_=0.108), with a strong linear relationship between group and PSI (contrast estimate=13.890, p<0.001).

After Bonferroni-correction for multiple comparisons, adolescent users with severe CUD had greater PSI-a scores than:

- adult users without severe CUD (t(93)=4.226, p<0.001, mean difference=15.244)
- adolescent controls (t(98)=3.944, p=0.002, mean difference=14.006)
- adult controls (t(99)=5.231, p<0.001, mean difference=18.523).

Also, adolescent users without CUD had marginally greater PSI scores than adult controls (t(120)=2.832, p=0.075, mean difference=9.942).

Qualitatively, the groups with the lowest PSI means were adult controls, adolescent controls, and adult users without severe CUD. Adolescent users without severe CUD and adult users with severe CUD had qualitatively similar means.

When a 2x2 ANOVA was conducted only within users, there were main effects of age-group (F(1,142)=4.273, p=0.041, η^2^_p_=0.029) and CUD-status (F(1,142)=4.525, p=0.035, η^2^_p_=0.031), but no interaction between age-group and CUD-status (F(1,142)=0.055, p=0.816, η^2^_p_<0.001). Neither the main effect of CUD-status, nor the main effect of age-group, were robust to the inclusion of covariates, including cannabis use frequency.

**Figure S3.** Psychotomimetic states inventory-adapted (PSI-a) mean scores, with distributions of data and 95% CIs for adult controls, adolescent controls, adult users without severe CUD (sCUD), adolescent users without severe CUD, adult users with severe CUD and adolescent users with severe CUD. Post-hoc comparisons are Bonferroni-corrected. ***p<0.001, **p<0.001, #p<0.1

## **6.3 Correlations between age-of-onset and mental health symptoms in adult users**

Within adult users, there were no significant correlations between age-of-onset and BDI (r(69)=0.005, p=0.966), BAI (r(69)=0.051, p=0.671) and PSI-a (r(69)=0.006, p=0.963). There was a negative trend association between age-of-onset and presence of severe CUD (r(69)=-0.210, p=0.080).

## **6.4 Cannabis type and THC concentration**

**Table S12**. Cannabis type most commonly used in the last 12 weeks by adolescent users and adult users from the timeline follow-back. Strong herbal cannabis refers to ‘skunk’, typically indoor-grown seedless cannabis, sinsemilla, or ‘high-grade’. Weak herbal cannabis refers to typically outdoor-grown cannabis, which is darker in colour and has seeds in it, and can be called ‘Thaiweed’, ‘brickweed’, ‘ditchweed’. Hash/resin refers to the brown solid which is made by compressing the resin glands/trichomes of the cannabis plant. The one ‘other’ was ‘THC oil’. The groups did not differ in their most common cannabis type (χ_3_^2^=3.866, p=0.276).

|  | Adolescent User | Adult User |
| --- | --- | --- |
| Strong herbal (i.e. ‘skunk’) | 69 (90.8%) | 59 (83.1%) |
| Weak herbal | 0 (0.0%) | 2 (2.8%) |
| Hash/resin | 7 (9.2%) | 9 (12.7%) |
| Other | 0 (0.0%) | 1 (1.4%) |
|  |  |  |

# **Section 7. Supplementary Discussion**

*CUD*

The mechanisms behind the heightened adolescent vulnerability to CUD should be explicated in future research. Previously, differential impacts of other alcohol and drug use on cannabis dependence in adolescents and adults have been found (Von Sydow et al., 2002), while the use of high-potency cannabis may be particularly deleterious in adolescents (Freeman & Winstock, 2015).

*Overall adolescent effects*

Apart from the relationship with CUD, our study does not support the theory that adolescence is a period in which the effects of cannabis use on mental health are more potent (Levine et al., 2017). However, consistent with existing research, adolescents, were found to have significantly higher levels of depression, anxiety and psychosis-like symptoms than adults, regardless of user-status (Twenge et al., 2019). In our study, adolescent status approximately doubled the odds of mild anxiety or depression.

*Frequency of cannabis use*

Also in opposition to our hypotheses, frequency of cannabis use was not significantly associated with severity of anxiety, depression or psychosis-like symptoms. This suggests that the impact of more frequent compared to less frequent cannabis use on mental health was not detectable in our sample of over 140 cannabis users. Larger, epidemiological studies have reported associations between cannabis use frequency and mental health outcomes (Henquet et al., 2005; Kuepper et al., 2011; Lev-Ran et al., 2014; Marconi et al., 2016; Troisi et al., 1998); however these comparisons often involve much less frequent use, for instance weekly vs. monthly comparisons. Our use of controls who had limited exposure to cannabis or to tobacco should have reduced the impact of unmeasurable confounding variables. It is possible that previous associations between infrequent cannabis use and poor mental health are attributable to unknown confounds.

*Further limitations and future research*

Although our adult users all began frequent cannabis use (>1 day/week) after the age of 18, many first tried cannabis before the age of 18. Furthermore, adolescent users predictably first tried cannabis earlier than adult users. Crucially, however, age-of-onset was not associated with depression, anxiety, or psychotic-like symptoms in the adult users. Hence, we can likely exclude the possibility that any early initial use of cannabis contributed to the adult users’ mental health symptoms. It is notable that some previous studies have found associations between mental health symptoms and an early age-of-onset in adult cannabis users (Hosseini & Oremus, 2019; Schubart et al., 2011; Stefanis et al., 2004), but we did not replicate this. In contrast, age-of-onset was marginally associated with the presence of severe CUD in adult users. A further limitation to consider is that we excluded participants receiving treatment for a mental health problem and those who had experienced psychosis. Adults are more likely than adolescents to receive treatment and to have already experienced psychosis. Thus our sample may be biased towards worse adolescent mental health, given problems are potentially more likely to be untreated (Sultan et al., 2018) or prodromal and not yet fully expressed.

There are three critical, but inter-related, time periods that should be considered in relation to studies investigating age and drug use:

- current age
- age-of-onset
- duration of use

In our study, younger age (i.e. being an adolescent) is associated with a younger age-of-onset and a shorter duration of use. Whereas in most studies previously conducted on this topic, participants are of varying ages and younger age-of-onset is associated with longer durations of use. This means it has often been difficult to disaggregate the impact of age-of-onset and duration of cannabis use. However, in our current study, one could argue that we are not able to disaggregate current age and age-of-onset; this might be relevant in the difference in rates of severe CUD between the adolescent and adult users. Moreover, our criteria allowed adult users to begin frequent cannabis use after age 18, which may still be considered ‘adolescence’. Hence, one could argue that some of our adult users began frequent cannabis use in late adolescence.

Although difficult to conduct, future studies could aim to include the following groups in order to parse out these potential differences:

- adolescent cannabis users
- adult cannabis users who initiated use at the same time as the adolescent cannabis users
- adult cannabis users who initiated use after a certain age (e.g. >18 or >21 years)
- adolescent controls
- adult controls

Furthermore, future studies may also wish to include groups of participants who began using cannabis in adolescence but stopped using at different points in life, to investigate if the consequences of adolescent and adult use are different, even following abstinence.

The impact of adolescent cannabis use may manifest itself more strongly later in life. Therefore, inclusion of adult users who initiated use as adolescents would be informative. However, our current aim was to investigate the association between current cannabis use and current depression and anxiety during adolescence and adulthood.

Our measurement of cannabis use frequency was likely to be accurate, because we conducted in-depth three-month timeline follow-back assessments (Robinson et al., 2014). However, future research should improve upon the measurement of cannabis potency and aim to collect more accurate assessments of cannabis quantity (Hindocha et al., 2018).

**References**

Adamson, S. J., Kay-Lambkin, F. J., Baker, A. L., Lewin, T. J., Thornton, L., Kelly, B. J., & Sellman, J. D. (2010). An improved brief measure of cannabis misuse: the Cannabis Use Disorders Identification Test-Revised (CUDIT-R). *Drug and Alcohol Dependence*, *110*(1–2), 137–143.

Bohn, M. J., & Babor, H. R. K. (1995). The Alcohol Use Disorders Identification Test (AUDIT): validation of a screening instrument for use in medical settings. *Journal of Studies on Alcohol and Drugs*, *56*(4), 423–432.

De Haan, L., Kuipers, E., Kuerten, Y., Van Laar, M., Olivier, B., & Cornelis Verster, J. (2011). The rT-18: a new screening tool to assess young adult risk-taking behavior. *International Journal of General Medicine*.

Freeman, T. P., & Winstock, A. R. (2015). Examining the profile of high-potency cannabis and its association with severity of cannabis dependence. *Psychological Medicine*, *45*(15), 3181–3189.

Gmel, G. (2001). Imputation of missing values in the case of a multiple item instrument measuring alcohol consumption. *Statistics in Medicine*. https://doi.org/10.1002/sim.837

Hawthorne, G., & Elliott, P. (2005). Imputing cross-sectional missing data: Comparison of common techniques. *Australian and New Zealand Journal of Psychiatry*. https://doi.org/10.1111/j.1440-1614.2005.01630.x

Henquet, C., Krabbendam, L., Spauwen, J., Kaplan, C., Lieb, R., Wittchen, H. U., & Van Os, J. (2005). Prospective cohort study of cannabis use, predisposition for psychosis, and psychotic symptoms in young people. *British Medical Journal*. https://doi.org/10.1136/bmj.38267.664086.63

Hindocha, C., Norberg, M. M., & Tomko, R. L. (2018). Solving the problem of cannabis quantification. *The Lancet Psychiatry*, *5*(4), e8.

Hosseini, S., & Oremus, M. (2019). The Effect of Age of Initiation of Cannabis Use on Psychosis, Depression, and Anxiety among Youth under 25 Years. In *Canadian Journal of Psychiatry*. https://doi.org/10.1177/0706743718809339

Kuepper, R., Van Os, J., Lieb, R., Wittchen, H. U., Höfler, M., & Henquet, C. (2011). Continued cannabis use and risk of incidence and persistence of psychotic symptoms: 10 Year follow-up cohort study. *BMJ*. https://doi.org/10.1136/bmj.d738

Lawn, W., Mokrysz, C., Petrilli, K., Lees, R., Borissova, A., Bloomfield, M., Freeman, T., & Curran, V. (2020). Teenagers, Compared to Adults, are More Vulnerable to the Psychotic-Like and Addiction-Forming Risks Associated With Chronic Cannabis Use. *Biological Psychiatry*, *87*(9), S227.

Levine, A., Clemenza, K., Rynn, M., & Lieberman, J. (2017). Evidence for the risks and consequences of adolescent cannabis exposure. *Journal of the American Academy of Child & Adolescent Psychiatry*, *56*(3), 214–225.

Lev-Ran, S., Roerecke, M., Le Foll, B., George, T. P., McKenzie, K., & Rehm, J. (2014). The association between cannabis use and depression: a systematic review and meta-analysis of longitudinal studies. *Psychological Medicine*, *44*(4), 797.

Marconi, A., Di Forti, M., Lewis, C. M., Murray, R. M., & Vassos, E. (2016). Meta-analysis of the association between the level of cannabis use and risk of psychosis. *Schizophrenia Bulletin*, *42*(5), 1262–1269.

NDTMS. (2019). *Alcohol and drug misuse and treatment statistics - GOV.UK*. https://www.gov.uk/government/collections/alcohol-and-drug-misuse-and-treatment-statistics

NHS-Digital. (2018). *Smoking, Drinking and Drug Use among Young People in England - NHS Digital*. https://digital.nhs.uk/data-and-information/publications/statistical/smoking-drinking-and-drug-use-among-young-people-in-england

NHS-Digital. (2019). *Statistics on Drug Misuse, England, 2019 - NHS Digital*. https://digital.nhs.uk/data-and-information/publications/statistical/statistics-on-drug-misuse/2019

Robinson, S. M., Sobell, L. C., Sobell, M. B., & Leo, G. I. (2014). Reliability of the Timeline Followback for cocaine, cannabis, and cigarette use. *Psychology of Addictive Behaviors*. https://doi.org/10.1037/a0030992

Schubart, C. D., Van Gastel, W. A., Breetvelt, E. J., Beetz, S. L., Ophoff, R. A., Sommer, I. E. C., Kahn, R. S., & Boks, M. P. M. (2011). Cannabis use at a young age is associated with psychotic experiences. *Psychological Medicine*. https://doi.org/10.1017/S003329171000187X

Shrive, F. M., Stuart, H., Quan, H., & Ghali, W. A. (2006). Dealing with missing data in a multi-question depression scale: A comparison of imputation methods. *BMC Medical Research Methodology*. https://doi.org/10.1186/1471-2288-6-57

Stefanis, N. C., Delespaul, P., Henquet, C., Bakoula, C., Stefanis, C. N., & Van Os, J. (2004). Early adolescent cannabis exposure and positive and negative dimensions of psychosis. *Addiction*. https://doi.org/10.1111/j.1360-0443.2004.00806.x

Sultan, R. S., Correll, C. U., Schoenbaum, M., King, M., Walkup, J. T., & Olfson, M. (2018). National patterns of commonly prescribed psychotropic medications to young people. *Journal of Child and Adolescent Psychopharmacology*, *28*(3), 158–165.

Troisi, A., Pasini, A., Saracco, M., & Spalletta, G. (1998). Psychiatric symptoms in male cannabis users not using other illicit drugs. *Addiction*, *93*(4), 487–492.

Twenge, J. M., Cooper, A. B., Joiner, T. E., Duffy, M. E., & Binau, S. G. (2019). Age, period, and cohort trends in mood disorder indicators and suicide-related outcomes in a nationally representative dataset, 2005–2017. *Journal of Abnormal Psychology*, *128*(3), 185.

Von Sydow, K., Lieb, R., Pfister, H., Höfler, M., & Wittchen, H. U. (2002). What predicts incident use of cannabis and progression to abuse and dependence? A 4-year prospective examination of risk factors in a community sample of adolescents and young adults. *Drug and Alcohol Dependence*. https://doi.org/10.1016/S0376-8716(02)00102-3
